# Recent Advances in Saffron Soil Remediation: Activated Carbon and Zeolites Effects on Allelopathic Potential

**DOI:** 10.3390/plants9121714

**Published:** 2020-12-05

**Authors:** Mahdieh Kheirabadi, Majid Azizi, Seyedeh Faezeh Taghizadeh, Yoshiharu Fujii

**Affiliations:** 1 Department of Horticultural Science, Ferdowsi University of Mashhad, Mashhad 91779-48974, Iran; mahdieh.kheirabadi@mail.um.ac.ir (M.K.); Taghizadehf971@mums.ac.ir (S.F.T.); 2 Pharmaceutical Research Center, Pharmaceutical Technology Institute, Mashhad University of Medical Sciences, Mashhad 91778-99191, Iran; 3 Department of International Environmental and Agricultural Sciences, Tokyo University of Agriculture and Technology, Fuchu, Tokyo 183-8538, Japan

**Keywords:** allelochemicals, natural herbicide, plant remnants, replanting, rhizosphere soil

## Abstract

Saffron (*Crocus sativus* L.) is a highly valuable plant. Iran provides nearly 90% of the world’s total saffron and is the biggest global producer. The allelopathic effects of saffron corm (SC) and saffron field soil (SFS) have been hypothesized to play an important role in replanting. Recently, adsorbent materials have been used to neutralize the effects of allelochemicals. These materials, including activated carbon and zeolite, have large surface areas, pore volumes, as well as tremendous adsorptive capacity and complex chemical and physical properties. In this study, three independent experiments were conducted. In the first test, the allelopathic effects of aqueous and methanolic extracts of SC remnant and 9-year-old SFS as well as filtered aqueous extract of soil were investigated. In the second assay, the effects of SC remnants and SFS with different ages (i.e., 4, 6, and 9 years old) in combination with adsorbents were examined on the germination and growth of lettuce (*Lactuca sativa* L.) seedlings by the sandwich method. In the third experiment, we examined the effects of SC remnants combined with adsorbents on lettuce growth parameters. Our results showed that the allelopathic effects of aqueous and methanolic extracts of SC remnant were significantly superior to those of 9-year-old SFS. The aqueous extract of SC remnant reduced the root length of lettuce by 50%. The use of activated carbon and zeolites significantly decreased the observed allelopathic effect. Moreover, lettuce growth in rhizosphere soil was significantly inhibited by SC remnant and SFS extracts. The allelopathic effects of SC remnants caused a growth imbalance between the shoot and roots. Based on biochemical analyses, using the adsorbents increased the carotenoid content and chlorophyll index of lettuce by 23.33% and 5.25%, respectively. Adsorbents may play a role in treating soils contaminated by allelochemicals.

## 1. Introduction

The genus *Crocus* belongs to the big plant family of Iridaceae and consists of about 85 flowering species. Autumn-flowering *C. sativus* is widely cultivated in Iran, India, Afghanistan, Greece, Morocco, Spain, and Italy. It is known as the most expensive spice in the world and has many beneficial health effects for humans due to the presence of three main bioactive compounds, including crocin, picrocrocin, and safranal. Picrocrocin and safranal are derived from carotenoid oxidation products and are responsible for the bitter taste and aroma, respectively. The demand for saffron is increasing worldwide because of its applications in cuisine, medicine, and cosmetics, as well as emerging beneficial health properties [1,2].

Saffron has widely been used as an herb in traditional medicine. Based on biological/pharmacological studies, saffron exhibits antioxidant, anticancer, cytotoxic, anti-inflammatory, and antibacterial/fungal effects. Because of its various chemically active moieties, saffron’s therapeutic efficacy has been examined in some brain diseases, such as Alzheimer’s disease, Parkinson’s disease, cerebral ischemia, and depression [3,4].

Saffron is a perennial crop with a longevity of up to 12 years, but the stands start self-thinning after 5 or 6 years, which is when the economic yield starts reducing. For re-planting saffron, many farmers remove the top 30-cm deep soil layer of old saffron fields and substitute it with new soil, but this method is expensive. Various methods have been proposed for removing and/or inhibiting allelopathic effects, but most of them are impractical because of high costs or subsequent secondary pollution [5,6]. There have been numerous studies on the planting and harvesting of saffron; however, re-planting problems are still ignored.

Allelochemicals are mainly produced as a result of secondary metabolism in plants and microorganisms (e.g., bacteria, viruses, algae, and fungi) and can markedly affect several biological processes in ecosystems and agro-ecosystems [7]. The production of these compounds depends on the existence of precursor molecules and the activation of specific genes which are required for the biosynthesis of allelochemicals. The activation of these genes is often influenced by environmental incentives. Inhibitory effects of safranal on the radicles of germinated experimental plants have already been reported [8,9,10,11]. Allelopathy is generally known as an interspecific phenomenon, but if the donor and receiver are the same, it is known as autointoxication [12].

Activated carbon refers to a group of carbon materials with high porosity and internal surface. The internal surface area represents a high adsorption capacity and reactivation capability which make these materials unique substances [13]. Due to the high porosity of activated carbon, organic molecules bond together through physical adsorption as well as ion binding [14]. Additionally, activated carbon with specific physical and chemical traits can induce soil remediation. Activated carbon is widely used to investigate root-mediated allelopathy in plants, especially in plant invasion biology, because it adsorbs and thereby neutralizes root exudates [6,15]. The current study aims to present an advanced method for the remediation of saffron field soil by activated carbon and zeolites.

## 2. Results

### 2.1. Saffron Corm (SC) and Soil Extracts Effects on Lettuce Seed Germination

#### 2.1.1. Saffron Aqueous Extract

**Figure 1:**
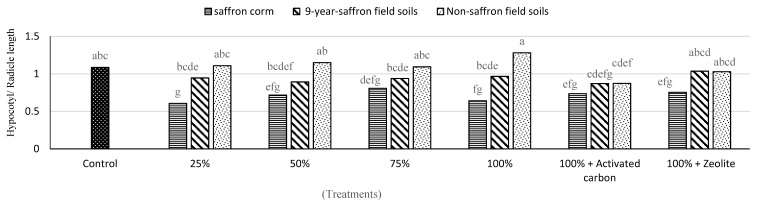
Effect of aqueous extracts of 9-year-old saffron corm, 9-year-old saffron field soil, and non-saffron field soil at different concentrations with activated carbon and zeolite on the ratio of hypocotyl length to radicle length of lettuce. Treatments connected by different letters on top are significantly different ((*p* ≤ 0.05).

The results of allelopathic activity of SC remnants and soil aqueous extracts on the germination of lettuce seeds showed that the highest inhibition was related to 9-year-old SC extract. Minimum germination percentages (82.0% and 86.5%) were obtained in SC extracts with 75% and 100% concentrations, respectively. The mean germination time of lettuce seeds significantly increased with respect to different concentrations of SC aqueous extract. The combination of activated carbon and SC remnants decreased mean germination time. However, no significant differences in the mean germination time were found between saffron field soil (SFS) and non-SFS extracts. Radicle and hypocotyl lengths did not show significant variations according to various concentrations of 9-year-old SFS and non-SFS extracts. The combinations of activated carbon and zeolite with 9-year-old SC remnants increased lettuce radicle and hypocotyl lengths (Table 1). Moreover, different concentrations of aqueous extract of 9-year-old SFS decreased the ratio of hypocotyl length to radicle length (Figure 1).

#### 2.1.2. Aqueous Extract of 9-Year-Old SFS and Adsorbents

**Figure 2:**
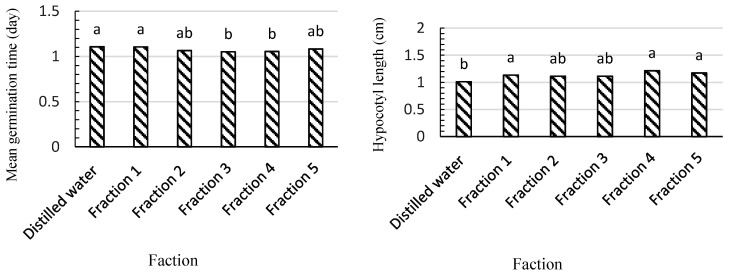
The effects of filtered aqueous extract of saffron field soil combined with activated carbon and zeolite on the mean germination time and hypocotyl length (cm) of lettuce seedlings. Fraction 1: 100% concentration (i.e., before passing through the adsorbent filter). Fraction 2: Quickly filtered extract. Fraction 3: The extract was filtered after 10 min. Fraction 4: The extract was filtered after 20 min. Fraction 5: The extract was filtered after 30 min. Treatments (bars) connected by different letters on top are significantly different (*p* ≤ 0.05).

Based on our results, filtered aqueous extracts combined with adsorbents did not show significant effects on the germination percentage of lettuce seeds, while radicle growth significantly decreased (Table 2). There were significant differences in the mean germination time and hypocotyl and radicle lengths between different treatment groups (Figure 2).

#### 2.1.3. Methanolic Extracts of 9-Year-Old SFS Combined with Adsorbents

The maximum germination percentage was observed in the control, the 9-year-old SFS, and the non-SFS groups. On the other hand, methanolic extract of 9-year-old SC delivered the highest inhibition of germination. Methanolic extracts of 9-year-old SC and 9-year-old SFS significantly increased the mean germination time. We also found that hypocotyl growth reduced in exposition to high concentrations of the extracts (Table 3).

### 2.2. Effects of SFS Extract on Lettuce via the Rhizosphere Soil Method

**Figure 3:**
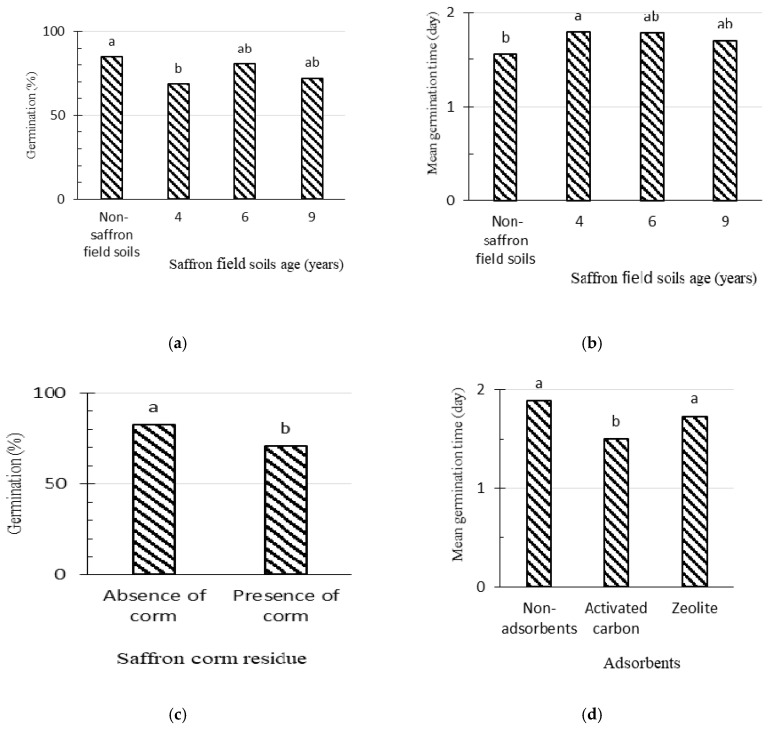
The effects of saffron field soil (**a**,**b**), saffron corm remnant (**c**), and adsorbents (**d**) on germination parameters of lettuce seeds via the rhizosphere soil method. Treatments (bars) connected by different letters on top are significantly different (*p* ≤ 0.05).

Our results showed no significant effects for SFS extract in terms of germination percentage. Nevertheless, SC remnant significantly reduced the germination percentage of lettuce seeds (Figure 3). Furthermore, SFS extract significantly increased mean germination time (Figure 3b), while activated carbon significantly decreased this variable and, consequently, increased germination rate (Figure 3).

**Figure 4:**
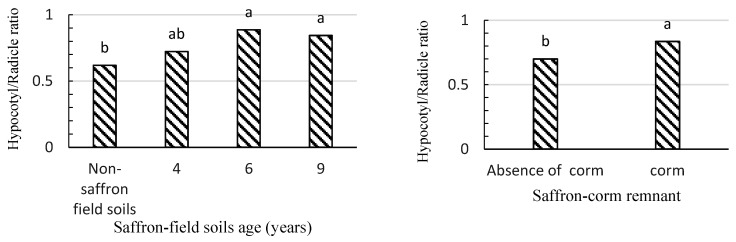
The effects of saffron field soil and saffron corm remnant on the ratio of hypocotyl length to radicle length of lettuce seedlings via the rhizosphere soil method. Treatments (bars) connected by different letters on top are significantly different (*p* ≤ 0.05).

Effects of SFS combined with either adsorbents or SC remnant significantly affected radicle and hypocotyl lengths. The minimum hypocotyl length was observed in 4-year-old SFS. Activated carbon in combination with SFS extracts significantly increased the radicle length (35.6%) (Table 4). Maximum hypocotyl length was related to 9-year-old SFS, which was associated with more pronounced imbalances in lettuce seedling growth parameters (Figure 4).

### 2.3. Effects of SC on Lettuce Growth

**Figure 5:**
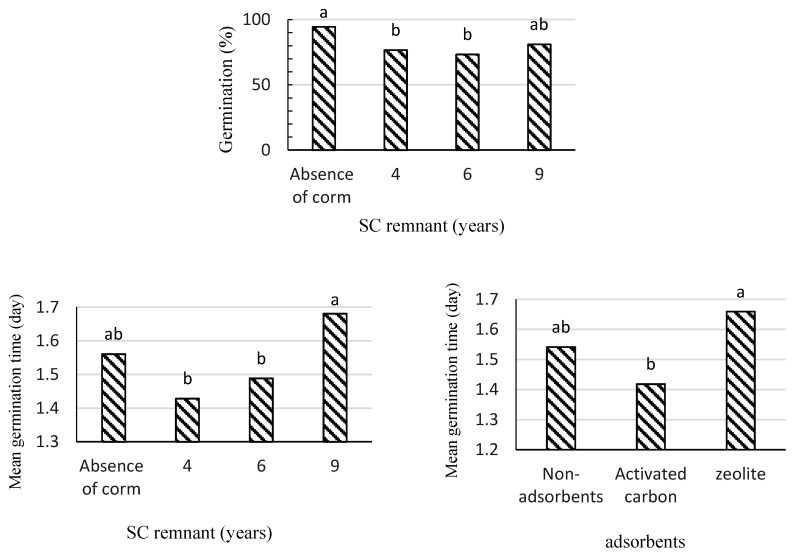
The effects of saffron corm remnants and adsorbents on the germination parameters of lettuce seeds via the sandwich method. Treatments (bars) connected by different letters on top are significantly different (*p* ≤ 0.05).

SC remnants and adsorbents did not show significant effects on the mean germination time. Activated carbon delivered the maximum ratio of radicle length to hypocotyl length, and the minimum ratio was obtained in 6-year-old SC (Table 5). Furthermore, there were significant differences in the effects of 6- and 4-year-old SC remnants on the germination parameters of lettuce seeds (Figure 5).

### 2.4. Effect of SC Remnants and Soil Extracts on Lettuce Growth in Greenhouse Condition

#### 2.4.1. Growth Parameters

**Figure 6:**
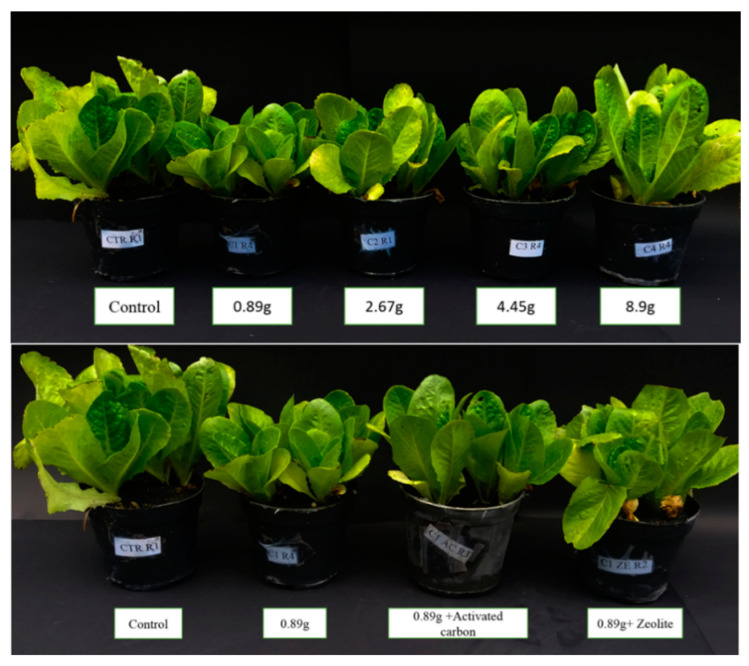
Effects of 9-year-old saffron corm remnants and adsorbents on lettuce growth parameters.

Significant effects were observed on root mass and leaves’ area in terms of the SC remnant and adsorbents. The maximum lettuce shoot height was related to the control (Figure 6). The combinations of adsorbents with SC remnants did not show significant effects on plant height. The allelopathic activity of SC remnants caused a growth imbalance between lettuce coleoptile and radicle (Table 6).

**Figure 7:**
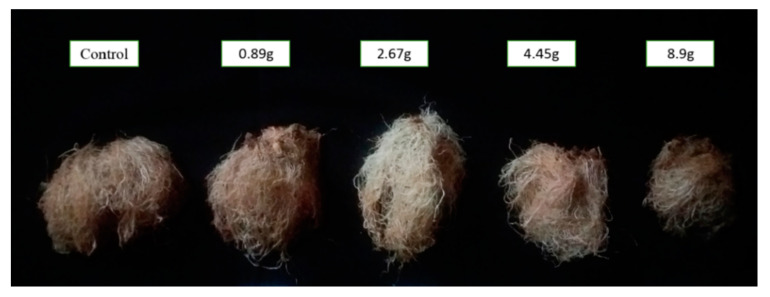
Effects of 9-year-old saffron corm remnants on lettuce root mass.

Using 9-year-old SC remnants significantly reduced lettuce root mass (Figure 7). Maximum root mass was obtained in control while adsorbents had no significant effects on root weight (Table 6).

#### 2.4.2. Physiological Parameters

Based on our results, although the combination of 9-year-old SC remnant and adsorbents did not show significant effects on electrolyte leakage and chlorophyll a content, they significantly affected stomata density. Table 6 shows the values of chlorophyll fluorescence in the light-adapted state (Fm′), variable fluorescence (Fv′), and the quantum yield of photosystem II (PSII) light-acclimated leaves (Fv′/Fm′).

##### Chlorophyll Index (SPAD Number)

The chlorophyll index (SPAD number) of lettuce seedling significantly increased in exposition to 9-year-old SC remnants. Applying activated carbon and zeolite also increased lettuce chlorophyll index (Table 7).

##### Chlorophyll and Carotenoid Contents

According to our results, 9-year-old SC remnants did not show significant effects on the total chlorophyll and chlorophyll b contents of lettuce. Moreover, the lettuce seedlings treated with zeolite showed the highest content of carotenoids (Table 7).

##### Chlorophyll Fluorescence

The effects of 9-year-old SC remnants on the fluorescence of light-acclimated leaves (F0′) showed that the maximum fluorescence was related to SC remnants (Table 7). Comparing SC remnants and adsorbents revealed that variable fluorescence (i.e., Fv′) was also related to SC remnants, showing a significantly different value from the fluorescence of activated carbon. Therefore, Fv′ and Fm′ were attributed to SC remnants and activated carbon, respectively (Table 6).

##### Stomata Density

In terms of lettuce leaf stomata features, no significant effects were observed for 9-year-old SC remnants nor for adsorbents on stomata density and stomata aperture length and width (Table 6).

#### 2.4.3. Allelopathic Activity in the Rhizosphere Soil Method

According to our findings, the allelopathic effects of SFS significantly reduced the growth of lettuce seedlings.

## 3. Discussion

Saffron (*C. sativus* L.) is one of the main plant sources of biologically active substances. The natural products derived from saffron are applied in ethnomedical, food, and cosmetic industrial processes [16,17]. Various factors (e.g., environmental stressors and allelochemicals) can influence the concentration and composition of secondary metabolites in plants and challenge their cultivation [18].

Based on the results of the present study, the aqueous extract of SC remnants showed significant effects on the percentage and mean time of germination, as well as the ratio of hypocotyl length to radicle length of lettuce seedlings. On the other hand, 9-year-old SFS aqueous extract did not show significant effects on the mentioned parameters.

Zhang and Yu reported that the soil extract of the Chinese fir tree significantly reduced seedling growth. Another research study showed that soil allelopathy, especially autotoxicity against pathogenic fungi, was a major factor in regulating production and nutrient cycling during continuous cultivation [19,20]. Hosseini and Rizvi showed no significant effects of SFS aqueous extract on the germination percentage of wheat, suggesting an important role for SC chemical leakage in modulating germination. They also reported that high concentrations of SC aqueous extracts (2- and 8-year-old) reduced the germination rate of wheat. Moreover, high levels of allelochemicals in SC remnants decreased wheat germination rate and seedling growth. In accordance, we found that the combinations of aqueous extracts with activated carbon and zeolite stimulated radicle and hypocotyl longitudinal growth [21].

According to our results, activated carbon and zeolite decreased the allelopathic activity of the assessed aqueous extracts and significantly increased lettuce seed germination and seedlings’ growth. Based on the report of Motoki et al., granular and powdered activated carbon elevated the uptake of asparagus allelochemicals [22]. According to another research study, rosemary extract did not affect the germination of lettuce seeds and other studied plants [23]. Several studies have reported that extracts of *Cyperus tuberosus*, *Alliaria petiolata*, and *Celastrus orbiculatus* exert no effects on the germination of seeds from lettuce and other plants [24,25].

Allelochemicals can change plants’ physiology, growth, and behavior or life cycle. Respiratory products, ATP content, seed germination, and seedling growth are delayed when plants are exposed to allelochemicals [26]. In contrast to optimal growing conditions, it seems that the rate of allelochemical production is higher under mineral deficiency, drought stress, and at low temperatures [27].

A previous study reported that asparagus extract inhibited plant growth, suggesting that difficulties in re-planting asparagus may be due to autotoxicity and pathogen accumulation [28]. The growth inhibitory effects of allelopathic plant extracts have also been reported by other researchers [29,30,31,32,33,34,35]. Low rates of cell division, elongation, and expansion were reported in the presence of allelochemicals [35,36].

Our results showed that activated carbon and zeolite reduced the allelopathic effects of 9-year-old SFS aqueous extract. It should be considered that adsorbent compounds are gradually saturated and may not be able to absorb allelochemicals. Therefore, the filtration of SFS extract via adsorbent compounds can play an important role in removing allelochemicals. Asao et al. showed that fine-activated carbon was more effective than coarse-activated carbon in improving plant growth, which was attributed to the higher surface area and adsorption capacity of the former [37].

The results of the present study showed that SC remnants significantly reduced the germination percentage of lettuce seeds. Lam et al. also reported that wheat root remnants reduced the biomass of the roots and shoot of the experimental plant. Accordingly, it has been suggested that autotoxicity might occur due to the accumulation of allelochemicals in remnants [28,38]. From an environmental point of view, because the allelopathic plants releasing allelochemicals into the environment are biodegradable, they cause less pollution, safeguard agronomic products, and alleviate environmental health problems. Therefore, these plants seem to be better options to replace chemical herbicides for weed management [39].

Based on our results, SC remnants reduced SPAD value. Various environmental stressors, including salinity, drought, organic pollutants, herbicides, UV light, and heavy metals, may cause physiological changes and increase oxidative stress in plants. In this regard, the levels of phytochemicals, chlorophyll, and secondary metabolites increase, and several enzymes are activated [18]. A reduction in chlorophyll content is observed in the plants exposed to environmental stressors, including herbicides and allelochemicals [40,41]. Allelochemicals enter the environment in a number of ways at different times, and the time of entry can alter their effects. Although chemicals with allelopathic activity may be present in many species, this presence does not mean that allelopathic effects will ensue. In this regard, allelochemicals can enter the environment by volatilization and may move through soil by leaching [42]. Other known entry pathways include (1) exudation and deposition on the leaf surface and subsequent washing off by rainfall; (2) exudation of volatile compounds from living green parts of the plant; (3) decay of plant residues (e.g., litterfall or dead roots), and (4) root exudation [43]. The exact chemical structures of allelochemicals need to be further investigated.

## 4. Materials and Methods

### 4.1. Soil and Plant Samples

Soil and corm samples were collected from perennial saffron fields in September 2018. Soil subjected to continuous saffron cropping and corm samples were collected from 4- (Sabzevar, Razavi Khorasan province, Iran, 36°12′45″ N 57°40′55″ E), 6-(Torbat-Heydariyeh, Razavi Khorasan province, Iran, 35°16′26″ N 59°13′10″ E), and 9-year-old (Mashhad, Razavi Khorasan province, Iran, 36°18′ N 59°36′ E) fields from the soil surface layer at 12–20-cm depth. Soil samples were randomly collected from three parts of each field (2 kg per part) and then mixed. The first experiment was conducted on 9-year-old SFS and SC remnants. Then, 4-, 6-, and 9-year-old SFS and SC remnants in combination with adsorbents were examined via the sandwich method in the second experiment. In the third and final experiment, we examined the effects of SC remnants in combination with adsorbents on lettuce growth parameters. In order to prepare non-SFS as control, soil samples were sieved (2-mm mesh) to remove remnants of plant organic materials. Accordingly, SC samples (500 g) were collected from different fields. Corms were cleaned and dried in an oven at 50 °C for 72 h, powdered, passed through a 0.5-mm mesh, and finally stored in a dark bottle at 4 °C until use.

Activated carbon is a granular water purifier (Clean pure UDF 10, Taiwan). Zeolite (clinoptilolite granular zeolite) was obtained from Semnan province, Iran. The chemical composition of zeolite included SiO_2_ = 65.90, Al_2_O_3_ = 11.20, Na_2_O = 2.10, K_2_O = 2.31, CaO = 3.20, Fe_2_O_3_ = 1.25, MgO = 0.52, LOI = 11.89, and SiO_2_/Al_2_O_3_ = 5.9 (mass percent). Lettuce seeds (Siaho cultivar, 90% germination rate) were purchased from Flat Tabriz Company, Iran. We used lettuce here due to its allelopathic sensitivity and uniform rapid growth [44]. Seeds were sterilized by 1% sodium hypochlorite and then washed three times with distilled water.

### 4.2. SC and Soil Extracts

#### 4.2.1. Aqueous Extract

The aqueous extract of 9-year-old SC was prepared based on the method described by Zackrisson and Nilsson (1992) [45]. Briefly, 50 g of corm powder was dissolved in 1 L of distilled water (5% solution) and shaken at 100 rpm for 48 h. The solution was filtered through a Whatman filter paper (No. 2) and diluted to 75%, 50%, and 25% (as stock solution). In order to remove allelopathic interactions, 2 g of powdered activated carbon or zeolite was added to 100 mL of SC aqueous extract and shaken at 100 rpm for 12 h.

The stock solutions of saffron and non-SFS were prepared in 1 L of distilled water. In order to evaluate the effect of activated carbon and zeolite on the allelopathic activity of the extracts, 2 g of activated carbon or zeolite was added to 100 mL of saffron and non-SFS (10% solution) aqueous extract of SFS. Then, each extract (through a Whatman No. 2 filter paper) and 50 lettuce seeds were placed into Petri dishes. Distilled water was used as a control. The procedure was conducted as a factorial experiment in a completely randomized design with five replications.

#### 4.2.2. Fractionation of Saffron Soil Aqueous Extract

**Figure 8:**
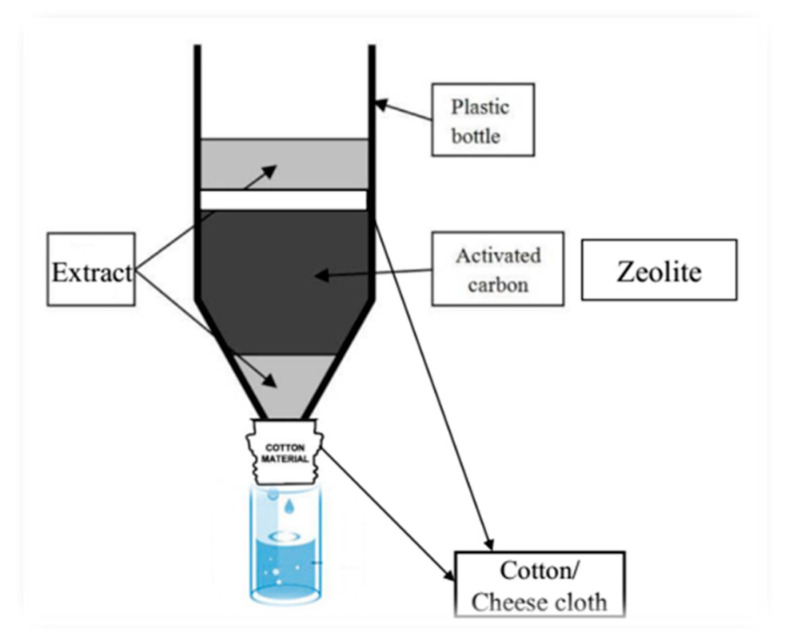
Fractionation of 9-year-old SFS aqueous extract after passing through activated carbon and zeolite.

In this experiment, different fractions of 9-year-old SFS aqueous extract were collected after passing through activated carbon or zeolite (Figure 8). The fractions included fraction #1: SFS extract (10% solution, non-filtered); fraction #2: the extract rapidly filtered through an absorbent layer; fraction #3: the extract filtered after 10 min; fraction #4: the extract filtered after 20 min; and fraction #5: the extract filtered after 30 min. Then, 2 mL of each extract (passing through a Whatman No. 2 filter paper) and 50 lettuce seeds were placed into Petri dishes. Distilled water was used as a control. The germination and growth rates of lettuce were evaluated by a factorial experiment in a completely randomized design with five replications.

#### 4.2.3. Methanolic Extracts

Thirty grams of SC remnant was extracted with 300 mL of methanol 70% (*v*/*v*) for 48 h according to the Kato-Noguchi method [46]. The extract was filtered with a Whatman No. 2 filter paper. The extraction process was repeated three times until the extracts became colorless. The solvents were removed by using a rotary evaporator (Heidolph Laborota 4000 efficient) at 40 °C. Various concentrations (10, 30, and 100 mg dry weight (DW) equivalent extract/mL) were prepared by dissolving dried extract in 0.3 mL of methanol. After filtration, the extracts were added to six-well multi-dishes. Methanol evaporated in a draft chamber, and then, the filter paper was moistened with 0.8 mL of 0.05% (*v*/*v*) Tween 20 solution. Tween 20 acts as a surfactant and does not inflict any toxic effects. After that, 500 g of each of 9-year-old SFS and non-SFS sample was extracted with 1 L of 70% methanol (*v*/*v*) for 48 h and then filtered (Kato-Noguchi, 2018). Dried extracts (10, 30, 100 mg dry weight of soil extract/mL) were filtered and added to six-well multi-dishes. Ten lettuce seeds were placed in each well. As the control group, seeds only moistened with 0.05% Tween 20 (*v*/*v*) solution (without extract) were used. The experiment was conducted as a factorial experiment in a completely randomized design with three replications [16,34].

### 4.3. Allelopathic Activity of SFS with Different Ages in the Rhizosphere Soil Method

This analysis was designed to investigate the allelopathic activity of SFS with different ages (4, 6 and 9 years old) either alone or in combination with SC remnants (9-year-old SCs) and activated carbon/zeolite by the soil rhizosphere method (Furubayashi et al., 2002; Fujii et al., 2005). Soil samples were collected from non-SFS fields. Culture medium was prepared by adding 5 mL of 75% agar cooled at 42 °C to a six-well multi-dish. After solidification of the agar surface, 3.2 mL agar (75%) was added to the first layer. Five lettuce seeds were separately placed on the agar surface. SC remnants and 50 mg of corm powders were mixed with soil. Adsorbent treatments were performed by adding 2 g of activated carbon/zeolite to 100 mL of agar solution, separately. The multi-dish was covered with parafilm and then incubated. According to Azizi et al., soil type and texture have no significant effects on the outcomes of this experiment [47]. The procedure was conducted as a factorial experiment with three factors in a completely randomized design (*n* = 3).

### 4.4. Allelopathic Activity of SC with Different Ages in the Sandwich Method

This experiment was designed to determine the allelopathic activity of SC remnants with various ages, either alone or in combination with the adsorbents via the sandwich method [44]. Here, 50 mg of powdered SC remnants was placed between two layers of agar 75% in 6-well multi-dishes (10-cm^2^ area per each dish). Five lettuce seeds were sown on the agar surface. Activated carbon and zeolite were prepared as previously described [10,41,48]. This assay was performed as a completely randomized design with three replications.

### 4.5. Allelopathic Activity of SC Remnants in Combination with Soil

In this experiment, lettuce seedlings were produced in a greenhouse and exposed to different weight ratios of 9-year-old SC remnants along with activated carbon/zeolite. The soil composition included garden soil (60%), coco peat (30%), and sand (10%). The soil was autoclaved at 121 °C for 15 min. Electrical conductivity (EC) and pH of the soil were 63.40 μS/cm and 7.2, respectively.

According to a previous study, the maximum yield is obtained by cultivating 50 saffron plants/m^2^ (i.e., 4 to 5 tons/ha) [49]. After seven years, corms and plants weaken, reducing the yield. SC remnants were selected as 0.2, 0.6, 1, and 2% *w*/*w* in each pot (10 × 10 cm^2^). Moreover, the amounts of activated carbon and zeolite were 3.99 g/pot (2400 kg/ha) and 4.45 g/pot (10 g/kg soil), respectively [37]. Four uniform lettuce seedlings (7-leaf stage) were transplanted in each pot. After four weeks, their growth and physiological parameters were measured. The experiment was set up in a factorial manner and a completely randomized design was used with 4 replications. The allelopathic activity of the pot soil was evaluated before and after lettuce cultivation via the rhizosphere soil method. For this experiment, multi-dishes, after sowing seeds, were kept in an incubator in the dark at 25 °C for three days. Then, seeds with the minimum root length (2 mm) were considered as germinated seeds, which were photographed to measure root and hypocotyl lengths using Image J software (1.52, National Institutes of Health, Wisconsin, MA, USA). Total germination percentage (Equation (1)), mean germination time (Equation (2)), radicle and hypocotyl lengths, and the ratio of radicle to hypocotyl length were calculated.

#### 4.5.1. Lettuce Seedling Growth Parameters

After four weeks of transplanting, leaf number and area, shoot height, shoot and root fresh and dry weights, and root mass were measured. Leaf area was measured by a WiNAReA-UT-11 leaf area meter.

#### 4.5.2. Physiological Parameters

- Chlorophyll fluorescence

A chlorophyll fluorescence assay was conducted at newly developed leaf tips (four plants per treatment) by a MINI-PAM Portable Chlorophyll Fluorometer. Measured parameters included minimum fluorescence light-acclimated leaves (F0′), maximum fluorescence light-acclimated leaves (Fm′), variable fluorescence (Fv′), and maximum quantum yield of PSII photosystems light-acclimated leaves (Fv′/Fm′).

- Chlorophyll index (SPAD number)

The soil-plant analysis development (SPAD) meter is a simple, rapid, non-destructive and low-cost apparatus that facilitate plant physiology research especially for determining N nutrition and chlorophylls content. SPAD level was recorded by a chlorophyll meter (SPAD-502 Konica Minolta, Tokyo) for newly developed leaves in the middle area (4 plants per treatment). A chlorophyll meter was used to determine the relative concentration of leaf chlorophyll based on the light passing through the leaf.

- Photosynthetic pigments (chlorophylls and carotenoids)

Photosynthetic pigments were measured according to the Lichthentaler method. Briefly, 0.5 g of fresh leaf tissue from 4 plants in each treatment was ground by a mortar by adding 10 mL of acetone (80%). The specimens were centrifuged (HERMLE-Z200A) at 2500 rpm for 20 min, and their absorptions were recorded by a CECIL CE 2502 2000 SERIES spectrophotometer at 663.2, 646.8, and 470 nm. Pigment concentrations were calculated using the following formulas:

Acetone, 80% (*v*/*v*):

Ca = 12.25A_663.2_ – 2.79A_646.8_ Cb = 21.50A_646.8_ – 5.10A_663.2_ Ca + b = 7.15A_663.2_ + 18.71A_646.8_ C x + c = (1000A_470_ – 1.82Ca − 85.02Cb)/198

where Ca is chlorophyll a; Cb is chlorophyll b; Ca + b is total chlorophylls and Cx + c is total carotenoids.

- Electrolyte leakage (EL)

EL was analyzed based on the method presented by Lutts et al. Accordingly, a leaf fragment (1 cm) from each plant was washed and placed in the tubes containing 10 mL distilled water (control). The tubes were shacked at 100 rpm (room temperature) for 24 h. The initial electrical conductivity (EC1) was measured by a conductivity meter (cc-511 ELMeIRON). The test tubes were then transferred into an autoclave where they were incubated at 121 °C for 20 min in order to destroy leaf cells. The final electrical conductivity (EC2) of cooled samples was also measured, and EL was calculated by the following equation:

EL = (EC1/EC2) × 100 (1)

#### 4.5.3. Stomata Characteristics

Leaf anatomical parameters, including stomata density and aperture characteristics (three parts per leaf), were analyzed by taking photographs under a light microscope (Olympus-BX41) at 40× and 100× magnification, respectively. The length and width of aperture were analyzed by Image J software (1.52a, National Institutes of Health, Wisconsin, MA, USA).

### 4.6. Lettuce Germination and Growth Characteristics

Total germination percentage, mean germination time, radicle and hypocotyl lengths, and radicle/hypocotyl length ratio were calculated as follows (Ronall et al.):

(2) $$Germination\;percentage\left(\%\right)=\frac{Number\;of\;germinated\;seeds}{Total\;number\;of\;seeds}\times 100$$

(3) $$Mean\;germination\;time\;\left(MGT\right)=\left[\frac{\sum_{i=1}^{k}n_{i}\cdot t_{i}}{\sum_{i=1}^{k}n_{i}}\right]\;$$

where *n_i_* is the number of seeds germinated at time *i*th; *K* is the last germination time.

### 4.7. Statistical Analysis

Statistical analyses were performed in SPSS software (version 23). Tukey’s Honest Significant Difference (HSD) test was used as the post hoc test (*p* ≤ 0.05). Graphs were plotted by Excel software (version 2016).

## 5. Conclusions

Our results revealed the allelopathic activities of SC remnants and SFS against lettuce germination and growth that were attributed to saffron allelopathic traits. Adsorbents, including activated carbon and zeolite, significantly improved lettuce growth parameters. The aqueous extract of SC remnant reduced lettuce root length by 50%, and lettuce growth in rhizosphere soil was significantly inhibited by SC remnants and SFS. The use of activated carbon and zeolite significantly decreased these allelopathic effects. Based on biochemical analyses, the adsorbents increased the carotenoid content and chlorophyll index of lettuce. Therefore, these adsorbents seem to be promising in ameliorating allelopathic effects and improving the physiological/biochemical quality of test plants. Further investigations are required to understand the active fraction and characterize the structures of the allelochemicals released by saffron. Our study confirms that absorbents may play a significant role in treating soils contaminated by allelochemicals.

## Figures and Tables

**Table 1:** Mean comparison of allelopathic effects of aqueous extracts from 9-year-old saffron corm, 9-year-old saffron field soil, and non-saffron field soil.

Concentration		Germination	(%)	Mean Germination Time (day)			Radicle Length (cm)			Hypocotyl Length (cm)		
	SC	SFS	Non-SFS	SC	SFS	Non-SFS	SC	SFS	Non-SFS	SC	SFS	Non-SFS
Control	95.20 ab			1.07 d	1.09 d	1.13 d	1.08 ef	1.19 d–g	1.12 e–g	1.17 def	1.12 f	1.18 def
25%	90.40 a–d	96.80 a	96.40 a	1.66 bc	1.08 d	1.08 d	2.66 a	1.23 d–g	0.98 ef	1.51 b	1.09 f	1.12 f
50%	84.40 cd	93.60 ab	96.00 a	1.89 ab	1.17 d	1.15 d	2.02 bc	1.20 d–g	1.07 ef	1.44 bc	1.13 f	1.16 def
75%	82.00 d	98.00 a	93.20 abc	1.95 a	1.09 d	1.09 d	1.67 cde	1.22 d–g	0.88 f	1.34 b–e	1.16 ef	1.12 f
100%	86.50 bcd	95.20 ab	97.60 a	1.66 bc	1.13 d	1.13 d	1.74 cd	1.48 c–f	1.34 d–g	1.11 f	1.27 c–f	1.17 def
100% + Activated carbon	91.60 abc	98.00 a	95.20 ab	1.44 c	1.13 d	1.18 d	2.51 ab	1.29 d–g	1.32 d–g	1.84 a	1.32 b–e	1.35 bcd
100% + zeolite	92.40 abc	96.80 a	96.80 a	1.67 bc	1.09 d	1.13 d	2.45 ab	1.19 d–g	1.12 e–g	1.84 a	1.12 f	1.18 def
Significance		*			**			**			**	
C.V		6.1%			32.2%			51.5%			21.4%	
LSD		5.09			0.13			0.30			0.10	

Numbers in each column followed by the same letter are not significantly different by Tukey’s test. (*p* ≤ 0.05). * indicates that the result is significantly different at *p* ≤ 0.05. ** indicates that the result is significantly different at *p* ≤ 0.01. NS: No significant difference. SC: Saffron corm. SFS: Saffron field soil. C.V: Coefficient of Variation. LSD: Least Significant Difference.

**Table 2:** Mean comparison of allelopathic effects of aqueous extracts of 9-year-old saffron field soil with activated carbon and zeolite on germination and seedling growth of lettuce.

Hypocotyl Length/Radicle Length	Radicle Length/Hypocotyl Length	Radicle Length (cm)	Different Fractions of Saffron Soils Extracts	Adsorbent
1.02 bc	0.98 abc	0.99 bc	-	Non-adsorbent (control)
0.89 c	1.11 a	1.26 a	Fraction 1	Activated carbon
0.91 c	1.09 ab	1.21 a	Fraction 2	
1.34 a	0.77 d	0.88 c	Fraction 3	
1.27 ab	0.79 cd	0.96 c	Fraction 4	
1.23 ab	0.81 cd	0.95 c	Fraction 5	
0.89 c	1.11 a	1.26 a	Fraction 1	Zeolite
1.14 abc	0.87 cd	0.97 c	Fraction 2	
1.10 abc	0.90 bcd	0.97 c	Fraction 3	
1.38 a	0.72 d	0.87 c	Fraction 4	
1.32 a	0.75 d	0.89 c	Fraction 5	
**	**	*		Significance
19.0%	18.1%	17.8%		C.V
0.16	0.12	0.13		LSD

Numbers in each column followed by the same letter are not significantly different by Tukey’s test. (*p* ≤ 0.05). Fraction 1: 100% concentration of extract (before the extract passed through the adsorbents filter). Fraction 2: The extract passed through the filter quickly. Fraction 3: The extract was filtered after 10 min. Fraction 4: The extract was filtered after 20 min. Fraction 5: The extract was filtered after 30 min. * indicates that the result is significantly different at *p* ≤ 0.05. ** indicates that the result is significantly different at *p* ≤ 0.01. NS: No significant difference.

**Table 3:** Effects of aqueous methanol extracts of 9-year-old saffron corm, 9-year-old saffron field soil, and non-saffron field soil on lettuce germination and seedling growth.

Sample	Concentration (mg Dry Weight Equivalent Extract/mL)	Germination (% of Control)	Mean Germination Time (Day)	Radicle Length (cm)	Hypocotyl Length (cm)	Radicle Length/Hypocotyl Length	Hypocotyl Length/Radicle Length
Control	NA	100.00 a	1.03 d	2.72 a	1.72 a	1.58 abc	0.63 b
	10 mg	81.70 a	1.86 bc	1.79 b	1.37 b	1.31 bc	0.76 b
9-year-old SC	30 mg	60.00 b	2.89 a	0.46 de	0.25 d	1.82 ab	0.55 b
	100 mg	NG	NG	NG	NG	NG	NG
	10 mg	100.00 a	1.50 c	0.64 cd	1.21 c	0.53 cd	1.88 a
9-year-old SFS	30 mg	98.30 a	1.98 b	0.80 c	0.31 d	2.59 a	0.40 bc
	100 mg	30.00 c	2.59 a	0.30 def	0.08 e	1.24 bcd	0.41 bc
	10 mg	100.00 a	1.60 bc	1.69 b	1.23 c	1.37 abc	0.72 b
Non-SFS	30 mg	83.30 a	1.94 b	0.41 de	0.32 d	1.29 bc	0.78 b
	100 mg	45.00 bc	1.83 bc	0.13 ef	0.10 e	1.28 bc	0.78 b
Significance		**	**	**	**	**	**
C.V		41.5%	45.8%	182%	90.3%	49.9%	68.4%
LSD		11.40	0.25	0.19	0.07	0.72	0.24

Numbers in each column followed by the same letter are not significantly different by Tukey’s test (*p* ≤ 0.05). ** indicates that the result is significantly different at *p* ≤ 0.01. NS: No significant difference. NG: Non-germinated.

**Table 4:** Mean comparison of allelopathic effects of saffron field soil, saffron corm remnant, and adsorbents on seedling growth of lettuce.

SC Remnant	Adsorbent	Saffron Field Soils Age (years)	Radicle Length (cm)	Hypocotyl Length (cm)	Radicle Length/Hypocotyl Length
Without saffron corm remnant		Non-SFS (control)	1.58 abc	0.88 a–f	1.78 abc
	No adsorbent	4	0.99 d–g	0.66 d–f	1.51 a–d
		6	1.14 c–g	0.94 a–f	1.22 cd
		9	1.13 c–g	1.03 a–e	1.08 d
		Non-SFS	2.14 a	0.99 a–f	2.15 a
	Activated carbon	4	2.05 ab	1.04 a–e	1.98 ab
		6	1.37 b-g	1.21 ab	1.22 cd
		9	1.44 b–e	1.06 a–d	1.37 bcd
		Non-SFS	1.78 abc	1.14 abc	1.52 a–d
	Zeolite	4	1.41 b–f	1.01 a–e	1.40 bcd
		6	2.04 ab	1.24 a	1.64 a–d
		9	1.03 d–g	0.69 def	1.47 a–d
With saffron corm remnant		Non-SFS	1.15 c–g	0.93 a–f	1.26 cd
	No adsorbent	4	0.79 efg	0.56 f	1.40 bcd
		6	0.90 d–g	0.79 b–f	1.14 cd
		9	0.70 g	0.61 ef	1.12 cd
		Non-SFS	1.11 c–g	0.62 def	1.79 abc
	Activated carbon	4	0.88 efg	0.72 c–f	1.23 cd
		6	1.06 d–g	0.94 a–f	1.13 cd
		9	1.01 d–g	0.91 a–f	1.10 cd
		Non-SFS	1.42 b–e	0.92 a–f	1.54 a–d
	Zeolite	4	0.72 fg	0.61 ef	1.18 cd
		6	0.96 d–g	0.99 a–f	0.95 d
		9	0.97 d–g	0.84 a–f	1.15 cd
Significance			**	**	**
C.V			47.3%	31.5%	26.2%
LSD			0.36	0.23	0.37

Numbers in each column followed by the same letter are not significantly different by Tukey’s test (*p* ≤ 0.05). ** indicates that the result is significantly different at *p* ≤ 0.01. NS: No significant difference. SC: Saffron corm. SFS: Saffron field soil.

**Table 5:** Mean comparison of allelopathic effects of saffron corm remnants and adsorbents on lettuce seedling growth.

Hypocotyl Length/Radicle Length	Radicle Length/Hypocotyl Length	Hypocotyl Length (cm)	Radicle Length (cm)	SC with Different Years	Adsorbent
0.54 c	1.85 a–d	0.79 ab	1.47 abc	Without corm	
0.49 c	2.06 ab	0.81 ab	1.65 ab	4	No adsorbent
0.63 abc	1.61 bcd	0.76 ab	1.23 bc	6	
0.69 abc	1.46 bcd	0.87 ab	1.27 abc	9	
0.41 c	2.39 a	0.82 ab	1.96 a	Without corm	
0.57 bc	1.74 a-d	0.80 ab	1.42 abc	4	Activated carbon
0.89 a	1.13 d	0.96 a	1.08 bc	6	
0.54 c	1.88 abc	0.56 c	1.07 bc	9	
0.56 c	1.87 abc	0.89 a	1.64 ab	Without corm	
0.66 abc	1.50 bcd	0.59 b	0.90 c	4	
0.86 ab	1.17 cd	0.84 ab	0.99 bc	6	Zeolite
0.69 abc	1.44 bcd	0.85 ab	1.22 bc	9	
*	*	*	*		Significance
35.8%	30.8%	26%	40.9%		C.V
0.18	0.38	0.23	0.37		LSD

Numbers in each column followed by the same letter are not significantly different by Tukey’s test (* *p* ≤ 0.05). NS: No significant difference. SC: Saffron corm.

**Table 6:** Mean comparison of interaction effects of allelopathic activity of 9-year-old saffron corm remnants and adsorbents on seedling growth of lettuce.

Stomata Number/Leaf Area (mm^−2^)	Fv′/Fm′	Fv′	Fm′	Leaves Area (cm^2^)	Roots Mass (cm^3^)	Plant Height (cm)	Saffron-Corm Remnant	Adsorbent
146.05 ab	0.67 cd	737.00 ab	1095.00 a	1720.00 a	3.33 a	14.00 a	0	Control
191.75 a	0.68 a–d	752.00 ab	1100.00 a	1198.00 bc	2.50 a–d	9.55 f	0.89	
137.98 ab	0.69 abc	769.00 a	1107.00 a	1394.00 abc	2.56 a–d	10.40 ef	2.67	Without adsorbent
195.99 a	0.67 abcd	799.00 a	1140.00 a	1192.00 bc	2.25 bcd	11.40 b–f	4.45	
131.44 ab	0.71 a	815.00 a	1142.00 a	1671.00 ab	2.17 cd	13.40 ab	8.90	
156.41 ab	0.64 d	669.00 b	983.00 b	1369.00 abc	2.63 a–d	11.00 def	0	
153.00 ab	0.67 a-d	760.00 a	1119.00 a	1487.00 abc	2.50 a–d	10.90 def	0.89	
119.66 b	0.69 abc	775.00 a	1118.00 a	1289.00 abc	2.42 bcd	9.88 ef	2.67	Activated carbon
154.23 ab	0.69 abc	772.00 a	1118.00 a	1315.00 abc	1.83 d	11.30 b–f	4.45	
192.22 a	0.70 abc	803.00 a	1145.00 a	1518.00 abc	2.08 cd	12.90 a–d	8.90	
179.50 ab	0.66 cd	745.00 ab	1088.00 a	1475.00 abc	2.56 a–d	12.0 a–e	0	
143.46 ab	0.67 bcd	752.00 ab	1115.00 a	1152.00 c	2.75 abc	9.39 f	0.89	
148.05 ab	0.67 a–d	727.00 ab	1071.00 ab	1183.00 bc	3.06 ab	9.66 f	2.67	Zeolite
121.55 b	0.70 ab	806.00 a	1135.00 a	1529.00 abc	2.25 bcd	11.20 c–f	4.45	
174.32 ab	0.70 abc	774.00 a	1093.00 a	1494.00 abc	1.83 d	13.20 abc	8.9	
**	*	*	*	*	*	**		Significance
21.3%	3.3%	6.2%	4.8%	17.4%	20.1%	14.1%		C.V
36.60	0	50.72	59.01	279.25	0.47	1.18		LSD

Numbers in each column followed by the same letter are not significantly different by Tukey’s test (* *p* ≤ 0.05). ** indicates that is significantly different at *p* ≤ 0.01. NS: No significant difference. Fm′: Maximum chlorophyll fluorescence in the light-adapted state. Fv′: Maximum level of variable fluorescence. Fv′/Fm′: Maximum and minimum quantum yields of photosystem II (PSII) photosystems light-acclimated leaves.

**Table 7:** Mean comparison of the simple effects of allelopathic activity 9-year-old SC remnants under different weight ratios and adsorbents on seedling growth of lettuce.

F0′	Carotenoids	Total Chlorophyll	Chlorophyll b	SPAD	Dry Weight of Shoot (g)	Fresh Weight of Shoot (g)	Number of Leaves	Dry Weight of Root (g)	Fresh Weight of Root (g)	Treatments
										SC remnant (g)
348.00 ab	1.20 a	0.97 b	0.39 b	22.30 b	0.54 a	5.97a	8.94 a	0.34 a	2.77a	0
357.00 a	1.16a	1.23 ab	0.53 ab	24.60 a	0.40 b	4.84 b	8.47 ab	0.30 a	2.71 a	0.89
342.00 abc	1.32 a	1.67 ab	0.66 ab	22.50 b	0.41 b	4.54 b	8.79 a	0.30 a	2.85 a	2.67
339.00 bc	1.13 a	1.93 a	0.81 a	21.30 bc	0.40 b	4.45 b	8.35 ab	0.21 b	1.95 b	4.45
329.00 c	1.31 a	1.67 ab	0.68 ab	20.00 c	0.40 b	4.61 b	7.83 b	0.21 b	1.96 b	8.9
**	NS	*	*	**	**	**	**	**	**	Significance
5.2%	-	65.6%	60.6%	11.1%	23.3%	22.8%	8.8%	30.9%	22.9%	C.V
12.79	-	0.60	0.23	1.61	0.06	0.73	0.53	0.05	0.31	LSD
										Adsorbent
342.00 a	1.06 b	1.31 a	0.68 a	21.40 b	0.44 a	5.09 a	8.43 a	0.29 a	2.69 a	Without adsorbent
347.00 a	1.29 a	1.43 a	0.59 a	23.00 a	0.43 a	4.94 a	8.50 a	0.27 a	2.37 b	Activated carbon
340.00 a	1.32 a	1.74 a	0.57 a	22.00 ab	0.41 a	4.62 a	8.50 a	0.25 a	2.28 b	Zeolite
NS	**	NS	NS	*	NS	NS	NS	NS	**	Significance
-	26.3%	-	-	11.1%	-	-	-	-	22.9%	C.V
-	0.17	-	-	1.50	-	-	-	-	0.33	LSD

Numbers in each column followed by the same letter are not significantly different by Tukey’s test (*p* ≤ 0.05). * indicates that the result is significantly different at *p* ≤ 0.05. ** indicates that the result is significantly different at *p* ≤ 0.01. NS: No significant difference.
